# Do we need two hammers in our toolbox? An empirical note about the potential redundancy of measuring subjective quality of life

**DOI:** 10.3389/fpubh.2025.1574241

**Published:** 2025-07-31

**Authors:** Otto R. F. Smith, Marit Knapstad, Leif Edvard Aarø

**Affiliations:** ^1^Department of Health Promotion, Norwegian Institute of Public Health, Bergen, Norway; ^2^Centre for Evaluation of Public Health Measure, Norwegian Institute of Public Health, Oslo, Norway; ^3^Department of Teacher Education, NLA University College, Bergen, Norway

**Keywords:** measurement, depression, quality of life, psychological distress, patient health questionnaire, Hopkins symptom checklist

## Abstract

**Background:**

Scales for the measurement of subjective quality of life (SQoL) and psychological distress are often used as if they measure different underlying concepts. This assumption is addressed in the present study by examining the discriminant validity between a set of items measuring SQoL and both the 2-item version of the Patient Health Questionnaire (PHQ-2) and the 5-item version of the Hopkins Symptom Checklist (HSCL-5).

**Methods:**

The present study is based on baseline data (*n* = 1,599) collected as part of the Students’ Psychological Health Over Time (SPOT) study, conducted among Norwegian university students. Data were examined by means of a bifactor analytical framework. The SQoL instrument was compared in separate analyses against the PHQ-2 and the HSCL-5.

**Results:**

Psychometric indices derived from the bi-factor model suggested that the SQoL instrument and the PHQ-2/HSCL-5 were essentially unidimensional. The overlap between scales was further confirmed by the finding that the associations between PHQ-2/HSCL-5 and a set of baseline correlates were similar to associations between the SQoL instrument and the same set of correlates.

**Conclusion:**

The SQoL instrument and the PHQ-2/HSCL-5 measure similar aspects for Norwegian university students. Combined with evidence from other studies, our findings suggest that using the SQoL instrument in addition to the PHQ-2 or HSCL-5 may be redundant.

## Introduction

Population-level statistics on subjective quality of life (SQoL; or wellbeing) are increasingly used as tools for guiding policies and policy decisions in many countries. It is suggested that in addition to using objective indicators on economy and living conditions, SQoL indicators should be included in all parts of policy development, among others, to determine what policy measures to prioritize, to examine the costs and benefits of different courses of action, to inform budget decisions, and to monitor and evaluate the effects of political choices (1–3). This trend is also evident in Norway, where the growing emphasis on quality-of-life (QoL) indicators underscores the need for valid and reliable measurement instruments.

In 2018, the Norwegian Directorate of Health (NDH) published a report with recommendations for a questionnaire-based tool for the measurement of quality of life (QoL) (4). The report distinguishes between subjective and objective QoL. Subjective QoL is about how life is experienced by the individual and includes cognitive, affective, and eudemonic aspects. Objective QoL is usually defined by more objective indicators of living conditions. In the NDH report, objective QoL includes aspects of life related to freedom, safety, health, community, and opportunities for self-development. In the report, the authors provide recommendations for measuring QoL based on existing instruments. For both subjective and objective QoL, a main list and a minimum list of items were derived. The main lists encompass 55 and 100 items, respectively, whereas the minimum lists encompass 12 and 11 items, respectively. The present study is particularly concerned with the minimum lists that are presented in this report.

The minimum list of subjective quality of life includes items measuring life satisfaction, positive and negative emotions, psychological functioning, and need satisfaction (Table 1). However, the minimum objective list also contains several items that are arguably subjective in nature, including two that assess depressive symptoms using the 2-item Patient Health Questionnaire (PHQ-2) (5), later replaced by the 5-item Hopkins Symptoms Checklist (HSCL-5) in a subsequent report (6). Although there is general agreement that subjective QoL and mental health are conceptually related (3), and some degree of overlap between the PHQ-2/HSCL-5 and subjective QoL items is expected, recent studies indicate that this overlap may be so substantial that the constructs are difficult to distinguish reliably and meaningfully (7–11).

**Table 1:** Minimum list subjective quality of life.

1. All things considered, how satisfied are you with your life as a whole these days? Give the answer on a scale from 0 to 10, where 0 means not satisfied at all and 10 means completely satisfied.
2. Overall, to what extent do you feel the things you do in your life are worthwhile? Give the answer on a scale from 0 to 10, where 0 means not meaningful at all and 10 means very meaningful.
Think about how you have been feeling for the past 7 days. To what extent were you….? Give the answer to a scale from 0 to 10, where 0 means you did not experience the feeling at all and 10 means you experienced the feeling to a very great extent. 3. Happy 4. Worried 5. Down or sad 6. Annoyed 7. Lonely 8. Engaged 9. Calm and relaxed 10. Anxious
How much do you agree with the statements below? Enter the answer on a scale from 0 to 10, where 0 means completely disagree and 10 means completely agree. 11. My social relationships are supportive and rewarding. 12. I actively contribute to the happiness and wellbeing of others.

This raises important questions about whether these instruments can justifiably be treated as measuring distinct constructs. To date, this issue has not been investigated in the context of the NDH’s minimum QoL list. Furthermore, the report recommends that the included items be analyzed largely in isolation, an approach that appears to assume adequate discriminant validity and unique informational value for each item.

However, discriminant validity, the extent to which a measure does not excessively overlap with other measures intended to assess different constructs, is often overlooked (12). This is particularly critical when QoL data are used to guide public policy at the national or local level. Therefore, the aim of the present study is to examine the discriminant validity between the subjective QoL indicators outlined in the NDH’s minimum list and commonly used measures of psychological distress, specifically the PHQ-2 and HSCL-5.

## Materials and methods

### Participants

The data are from the students’ psychological health over time (SPOT) study. The Regional Committee for Medical and Health Research Ethics in South-East Norway (no. 2019/1325) approved the study. All students who attended the University of Bergen (UoB) in January 2020 with Norwegian as their native language were eligible to participate (*N* = 15,816). The UoB provided the National Centre of Research Data (NSD) with contact information from all their students at the time. Of the total number of invited students, 4,823 agreed to participate and to complete the baseline questionnaire (response rate = 30.5%). Participants were randomized to different versions of the questionnaire, and approximately one-third of participants were asked to complete the version with the QoL items (*n* = 1,599).

### Primary measures

Subjective QoL was assessed based on a list of 12 items proposed in the NDH report (4) (The Minimum List, see Table 1). The items are intended to cover three dimensions: cognitive (item 1), positive and negative affect (items 3–10), and eudemonic (items 2, 11, 12). Responses for all 12 questions were coded from 0 (i.e., not at all) to 10 (i.e., to a very high degree). To aid interpretation, positively phrased questions were recoded such that a low score of all items represented high QoL, and a high score represented low QoL.

Our initial approach was to analyze the factor structure of the minimum list by means of exploratory factor analysis, in which the number of factors (k) retained was determined by means of a parallel analysis. Although parallel analyses generally perform well, there is some evidence that the number of factors may, in some instances, be underestimated by one factor (13). Factor solutions with k and k + 1 factors were therefore examined for interpretability.

The parallel analysis suggested a two-factor solution, although this was associated with a poor model fit (CFA = 0.892, RMSEA = 0.128, SRMR = 0.048) and too many significant cross-loadings. Although the model fit was better for the three-factor solution (CFA = 0.946, RMSEA = 0.104, SRMR = 0.032), there were still four items with comparatively high cross-loadings. See Supplementary Table A1 for more details on the factor solutions.

Based on these results, we chose to adopt another approach based on the analysis guidance provided in the NDH report, which recommends analyzing items 1, 2, 11, and 12 separately and calculating mean scores for the positive emotion items and for the negative emotion items. These six indicators were included in a one-factor model to represent subjective quality of life, but this again resulted in poor model fit (CFA = 0.863, RMSEA = 0.171, SRMR = 0.070). Nonetheless, in this case, only one modification index stood out and suggested that the correlation between items 11 and 12 was not sufficiently explained by the latent factor (modification index = 292.838). As both items tap into social aspects of subjective quality of life, we decided to calculate their mean score and use it as a single indicator instead. The model with the five indicators provided a more acceptable model fit and was therefore used in subsequent analyses (CFA = 0.983, RMSEA = 0.073, SRMR = 0.021, see also Supplementary Table A2). Cronbach’s alpha for the 5-indicator version of the SQoL questionnaire was 0.86 in the present study.

Psychological distress was measured by means of the 2-item versions of the Patient Health Questionnaire (PHQ-2). This abbreviated version of the original PHQ-9 comprises the two main DSM-IV criteria for major depression (5, 14). The abbreviated measures correlate strongly with their original counterpart. Participants were asked how often they were bothered by each of the symptoms during the last week on a scale from 0 (not at all) to 6 (all the time). A 7-point scale instead of the original 4-point scale was chosen to increase measurement reliability (15), which was important in light of the original aim of the SPOT study. Cronbach’s alpha was 0.86 for PHQ-2.

The other measure of psychological distress was the 5-item version of the HSCL. This abbreviated version includes five items, each rated on a Likert scale from 1 (“not bothered”) to 4 (“very bothered”). Participants indicate how much they have been bothered by each symptom during the past 2 weeks (6).

### Correlates of subjective QoL and psychological distress

The Big Five Inventory (BFI) was used to assess three of the five personality traits: extraversion, conscientiousness, and neuroticism (16, 17) and was included due to their established associations with anxiety, depression, and quality of life (18). The abbreviated version of the BFI was used for extraversion (four items each) (19), whereas the full version was used for conscientiousness (nine items) and neuroticism (eight items). It has been shown that both the full and abbreviated versions of the BFI have acceptable psychometric properties (16, 17). The response scale ranged from 0 (disagree strongly) to 4 (agree strongly). Subscale scores are presented as average sum scores with a 0–4 range. In the present study, Cronbach’s alpha was 0.82 for extraversion, 0.80 for conscientiousness, and 0.87 for neuroticism.

Other variables that were used as correlates of subjective quality of life and psychological distress were: age in years, gender (m/f), parental educational level (low, medium, and high), having a partner (y/n), self-rated health (very good, good, not so well, and bad), relative effort in high school (0 – much less effort to 4 – much more effort), and average grade in high school (0–6).

### Statistics

**Figure 1:**
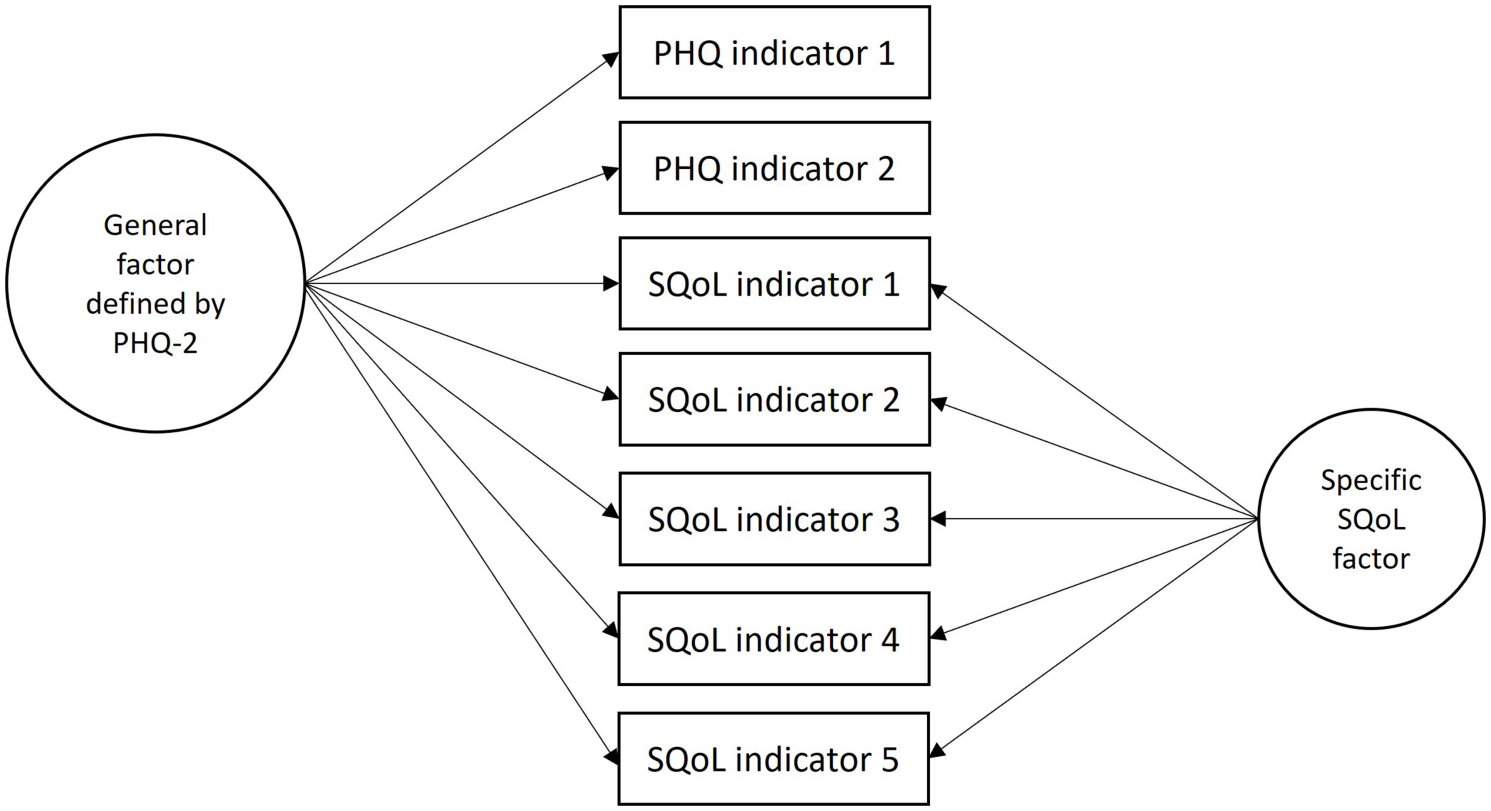
Example of a bifactor (S-1) model.

When estimating the bifactor model, we included one general factor consisting of all items of either the PHQ-2 or HSCL-5 and the previously defined five indicators of subjective QoL, and only one specific factor consisting of the subjective QoL items. This is called a ‘Bifactor-(S-1) Model’ and has been described by Eid et al. (20). In our context, the purpose of this procedure is to produce a well-defined psychological distress factor which captures all the reliable variance in the PHQ/HSCL items together with the part of the common subjective QoL variance that it shares with the PHQ/HSCL scale (see Figure 1). In this way, the specific subjective QoL factor will consist of the reliable variance that is unique to the subjective QoL scale (orthogonal residual factor).

A number of psychometric indices were derived: global omega (*ω*), omega hierarchical (ωH), omega subscale (ωS), omega hierarchical subscale (ωHS), explained common variance (ECV), percent of uncontaminated correlations (PUCs), average relative parameter bias (ARPB), and individual explained common variance (IECV) (21).

Different guidelines exist for assessing whether multidimensionality is severe enough to disqualify an instrument as primarily unidimensional. When ECV is above 0.80, relative bias will generally be lower than 5%, and when ECV is above 0.70, relative bias will generally be lower than 10%. Similar cutoffs can be applied to PUCs. However, when PUC values become lower, general ECV values are less important in predicting bias related to forcing a unidimensional model to multidimensional data. That is, when PUC values are lower than 0.80, general ECV values are greater than 0.60, and ωH > 0.70, the multidimensionality is not sufficiently large to reject the interpretation of the instrument as primarily unidimensional. For ARPB, a value below 15% is considered acceptable in this regard (22–24).

To validate the findings of the bifactor model, we also examined bivariate correlations between average sum scores of PHQ-2, HSCL-5, subjective QoL, and the five separate subjective QoL indicator scores on the one hand, and a relatively broad selection of variables described earlier in the method section on the other hand. If the PHQ and the subjective QoL items primarily measure the same construct, we would expect their correlations with other variables to be similar.

## Results

### Sample characteristics

The student sample had an average age of 24.1 years, and 69% of them were women. Approximately half of the participants reported having a partner, and the majority had parents with higher education (see Table 2). Overall, participants reported good health, relatively low levels of psychological distress, and high QoL levels.

**Table 2:** Sample characteristics.

Variable	
Female sex, % (n)	69.0 (1093)
Age, mean (SD)	24.1 (4.7)
Higher education mother, % (n)	68.9 (1018)
Higher education father, % (n)	63.1 (882)
Having a partner, % (n)	47.0 (750)
Self-rated health, mean (SD)	0.9 (0.8)
Extraversion, mean (SD)	2.3 (1.0)
Neuroticism, mean (SD)	1.9 (0.9)
Conscientiousness, mean (SD)	2.6 (0.7)
Relative high school effort, mean (SD)	1.8 (1.1)
High school grade, mean (SD)	4.8 (0.7)
PHQ-2, mean (SD)	1.7 (1.6)
HSCL-5, mean (SD)	2.0 (0.8)
Subjective Quality of Life, mean (SD)	3.4 (1.7)
SQoL indicator 1, overall QoL, mean (SD)	3.2 (2.1)
SQoL indicator 2, meaning in life, mean (SD)	3.4 (2.5)
SQoL indicator 3, positive emotions, mean (SD)	4.1 (2.0)
SQoL indicator 4, negative emotions, mean (SD)	3.8 (2.2)
SQoL indicator 5, social integration, mean (SD)	2.4 (2.0)

### Findings from the bifactor (S-1) model

We found support for the unidimensionality of the PHQ-2 in combination with the SQoL (CFA = 0.983, RMSEA = 0.073, SRMR = 0.024, see also Supplementary Table A3). Both ECV and OmegaH were higher than 0.80, whereas OmegaHS was lower than 0.30. The ARPB was also below the acceptable upper limit of 15% bias (see Table 3).

**Table 3:** Omega coefficients and related indices across bifactor (S-1) models.

Index	G-factor; PHQ-2	G-factor; HSCL-5
Omega	0.920	0.938
OmegaH	0.804	0.839
OmegaHS_QoL_	0.225	0.297
PUCs	0.524	0.756
ECV	0.807	0.759
ARPB	0.146	0.077
IECV SQoL indicator 1	0.689	0.587
IECV SQoL indicator 2	0.574	0.478
IECV SQoL indicator 3	0.737	0.637
IECV SQoL indicator 4	0.999	0.996
IECV SQoL indicator 5	0.486	0.348
Model fit		
RMSEA	0.086	0.077
CFI	0.977	0.963
SRMR	0.024	0.037

Model fit for the bifactor (S-1) model with HSCL-5 defining the general factor did not yield an acceptable model fit due to an unexplained residual correlation between HSCL-item 1 (feeling fearful) and HSCL-item 2 (nervousness or shakiness inside; CFA = 0.933, RMSEA = 0.100, SRMR = 0.047). As both items cover the affective aspects of anxiety, we added these as a second specific factor to the bifactor (S-1) factor. This yielded a more acceptable model fit (CFA = 0.963, RMSEA = 0.077, SRMR = 0.037, see also Supplementary Table A3), and effectively changed the meaning of the general factor to a pure depression factor, similar to the previous model based on PHQ-2. As shown in Table 3, support for unidimensionality was found in this revised model as well (ECV = 0.759, OmegaH = 0.839, OmegaHS = 0.297, and ARPB = 0.077).

The SQoL indicator that shared the least overlap with the PHQ/HSCL scales was indicator 5 on social integration, as indicated by relatively low IECV levels, which makes sense given that this indicator fits conceptually less well with the other indicators of the SQoL instrument and may rather be a determinant than an indicator of SQoL.

### Correlations with other variables

The correlations of the outcome variables with the included correlates generally supported the findings derived from the bi-factor models (Table 4). The patterns of correlations between the PHQ-2, HSCL-5, and the SQoL were very similar. There was somewhat more variation between the PHQ-2, HSCL-5, and the individual SQoL items, but the differences were generally small.

**Table 4:** Correlates of psychological distress and subjective QoL*.

	PHQ-2	HSCL-5	SQoL	SQoL 1	SQoL 2	SQoL 3	SQoL 4	SQoL 5
Sex	−0.058	−0.201	−0.009	0.021	0.046	−0.053	−0.152	0.086
Age	0.036	0.014	0.053	0.064	−0.053	0.049	0.077	0.060
Education mother	−0.101	−0.092	−0.142	−0.122	−0.083	−0.137	−0.098	−0.113
Education father	−0.055	−0.081	−0.108	−0.078	−0.058	−0.096	−0.086	−0.079
Having a partner	−0.106	−0.053	−0.172	−0.209	−0.155	−0.097	−0.074	−0.133
Self-rated health	0.473	0.494	0.488	0.478	0.368	0.406	0.413	0.264
Extraversion	−0.309	−0.297	−0.417	−0.330	−0.290	−0.342	−0.299	−0.403
Neuroticism	0.555	0.734	0.623	0.511	0.419	0.535	0.697	0.311
Conscientiousness	−0.291	−0.218	−0.354	−0.320	−0.365	−0.244	−0.205	−0.254
High school effort	−0.060	0.078	−0.074	−0.074	−0.123	−0.030	0.060	−0.117
High school grade	−0.129	−0.115	−0.184	−0.167	−0.200	−0.109	−0.090	−0.156

*Higher PHQ and HSCL scores indicate higher levels of psychological distress. Higher SQoL scores indicate lower levels of quality of life.

## Discussion

Psychometric indices derived from the bifactor models indicated that the SQoL instrument was essentially unidimensional in relation to both the PHQ-2 and the HSCL-5. This overlap was further supported by the finding that the associations between the PHQ-2 and HSCL-5 and a broad range of baseline correlates closely mirrored those observed for the SQoL instrument. Together, these results suggest a lack of discriminant validity between the SQoL questionnaire recommended by the NDH and commonly used measures of psychological distress in a sample of Norwegian university students, particularly in relation to items assessing depressive symptoms. Although it is well known that measures of psychological distress and SQoL are correlated, the present findings indicate that the degree of overlap may be greater than typically acknowledged.

Our findings align with previous studies that have explored the overlap between related constructs, such as depressive symptoms and positive mental wellbeing (7, 10). A common issue among these studies is that, while the constructs differ theoretically, they often show substantial empirical overlap. In practice, this means that using these measures frequently yields similar information, despite being framed or labeled differently.

The limited, unique, and independent information provided by the PHQ-2/HSCL-5 and the subjective quality of life (SQoL) scales may pose a challenge, especially as many governments increasingly aim to incorporate quality of life metrics as overarching targets in political and policy decision-making (2, 4). For these metrics to function effectively, it is essential that the instruments used capture distinct and meaningful aspects of wellbeing.

The National Data Health (NDH) report’s recommended minimum list includes the PHQ-2/HSCL-5 as a measure of objective quality of life, alongside a separate list for subjective quality of life, which was the focus of the present study. However, the PHQ-2/HSCL-5 is inherently subjective in nature, raising questions about its classification as an indicator of objective QoL. Moreover, our findings suggest that the recommended SQoL measures do not contribute substantial unique variance beyond what is already captured by the PHQ-2/HSCL-5, rendering their added value questionable in practice.

A commonly held belief is that adding a positive dimension to the traditionally deficit-oriented approach to mental health (i.e., focusing on wellbeing in addition to distress) will yield substantially new and complementary information (25, 26), but this assumption may be more theoretical than empirical. A general population study by Bohnke and Croudace provided compelling evidence that the concept of positive mental wellbeing measured largely the same as a psychological distress measure. Interestingly, they found that these instruments predominantly assessed states *below* the population mean rather than *above* it (as evidenced by test information curves (7)). The latter would be required to potentially extend the continuum to the positive side of mental wellbeing. Whether this also applies to subjective quality-of-life instruments more broadly remains to be seen in future studies.

## Strengths and limitations

Key strengths of this study include the relatively large sample size, the robust analytical approach, and the explicit focus on discriminant validity, an important yet often overlooked aspect of measurement research. A limitation is that the SQoL scale was not originally developed for psychometric scaling, which presented challenges in applying factor-analytic techniques. As described, we aggregated the positive emotion, negative emotion, and social integration items into mean scores. This data reduction likely led to some loss of information and may have attenuated some of the unique variance of the SQoL measure.

To address this, we also tested a model using all 12 items as separate indicators in a four-factor structure, thereby preserving the potential multidimensionality of the SQoL instrument. Although not presented in the results section, this alternative model produced findings that were largely consistent with those reported here, suggesting that item aggregation did not substantially influence the results of the current study (see Supplementary Appendix B).

Another limitation is the restricted generalizability of our findings. The study sample consisted exclusively of university students, and the results may not extend to broader populations or different quality-of-life instruments. Cultural variability is another important consideration. Norms surrounding emotional expression and mental health stigma may influence how individuals respond to subjective wellbeing measures, potentially yielding different results across cultural contexts.

Finally, we used a preliminary version of the recommended questionnaire-based QoL instrument (4), rather than the updated 2023 version (2). However, there is a considerable overlap between the two. Items 1–10 from Table 1 remain unchanged, whereas items 11–12 have been replaced by mastery-related items (e.g., “I have little control over what happens to me,” “When faced with problems in life, I often feel helpless”). These new items appear to better reflect subjective QoL, but may, as a result, have greater overlap with distress measures such as the PHQ-2/HSCL-5 compared to items 11–12 in the present study.

## Conclusion

Our findings suggest that there may be a lack of discriminant validity between measures of subjective quality of life and psychological distress, in particular symptoms of depression. We conclude that the use of general subjective quality of life instruments may therefore be redundant in the presence of PHQ-2 or HSCL-5. More research is needed to address the issue of discriminant validity more broadly, both from a conceptual and a measurement point of view.

## Data Availability

The datasets generated and/or analyzed during the current study are not publicly available due to ethical restrictions and personal data protection but are available from the corresponding author on reasonable request. Requests to access the datasets should be directed to Robert Smith, robert.smith@fhi.no.
