# Supplementary material for: Do we need two hammers in our toolbox? An empirical note about the potential redundancy of measuring subjective quality of life
Source: Front Public Health. 2025 Jul 31;13:1574241. doi: 10.3389/fpubh.2025.1574241 (PMC12350479; doi:10.3389/fpubh.2025.1574241)
Supplement: Supplementary file 1 [file Supplementary_file_1.docx]

**Appendix A. Standardized estimates for various factor models.**

*Table A1. 2- and 3-factor solutions for the minimum list of subjective quality of life (EFA).*

*2-factor model:*

GEOMIN ROTATED LOADINGS (* significant at 5% level)

1 2

________ ________

ITEM1R 0.809* 0.135*

ITEM2R 0.762* 0.005

ITEM3R 0.826* 0.009

ITEM4 -0.066* 0.898*

ITEM5 0.310* 0.617*

ITEM6 0.084* 0.476*

ITEM7 0.425* 0.328*

ITEM8R 0.661* -0.132*

ITEM9R 0.366* 0.344*

ITEM10 0.007* 0.877*

ITEM11R 0.651* -0.059

ITEM12R 0.689* -0.148*

RMSEA (Root Mean Square Error Of Approximation)

Estimate 0.128

90 Percent C.I. 0.122 0.135

CFI/TLI

CFI 0.892

TLI 0.835

SRMR (Standardized Root Mean Square Residual)

Value 0.048

*3-factor model:*

GEOMIN ROTATED LOADINGS (* significant at 5% level)

1 2 3

________ ________ ________

ITEM1R 0.812* 0.142* 0.013

ITEM2R 0.798* -0.003 -0.030

ITEM3R 0.791* 0.031 0.047*

ITEM4 -0.102* 0.917* 0.014

ITEM5 0.324* 0.618* -0.045*

ITEM6 0.071* 0.484* 0.001

ITEM7 0.289* 0.386* 0.173*

ITEM8R 0.672* -0.129* -0.014

ITEM9R 0.338* 0.360* 0.023

ITEM10 0.004 0.879* -0.026

ITEM11R 0.001 0.172* 1.038*

ITEM12R 0.362* -0.018* 0.461*

RMSEA (Root Mean Square Error Of Approximation)

Estimate 0.104

90 Percent C.I. 0.096 0.111

CFI/TLI

CFI 0.946

TLI 0.892

SRMR (Standardized Root Mean Square Residual)

Value 0.032

*Table A2. Factor solution of the 1-factor SQoL model.*

STDYX Standardization

Two-Tailed

Estimate S.E. Est./S.E. P-Value

SQoL BY

SQoL1 0.926 0.009 106.920 0.000

SQoL2 0.783 0.016 49.714 0.000

SQoL3 0.765 0.016 47.578 0.000

SQoL4 0.643 0.019 33.729 0.000

SQoL5 0.589 0.022 27.275 0.000

RMSEA (Root Mean Square Error Of Approximation)

Estimate 0.073

90 Percent C.I. 0.054 0.094

Probability RMSEA <= .05 0.025

CFI/TLI

CFI 0.983

TLI 0.966

SRMR (Standardized Root Mean Square Residual)

Value 0.021

*Table A3. Bifactor solutions for PHQ-2 and HSCL-5.*

*PHQ-2*

STDYX Standardization

Two-Tailed

Estimate S.E. Est./S.E. P-Value

GEN BY

SQoL1 0.755 0.017 44.295 0.000

SQoL2 0.607 0.021 28.459 0.000

SQoL3 0.664 0.020 33.521 0.000

SQoL4 0.768 0.014 54.689 0.000

SQoL5 0.423 0.027 15.734 0.000

PHQ1 0.822 0.013 61.914 0.000

PHQ2 0.926 0.011 87.625 0.000

SQoL BY

SQoL1 0.507 0.028 18.265 0.000

SQoL2 0.523 0.026 19.920 0.000

SQoL3 0.397 0.027 14.443 0.000

SQoL4 0.028 0.030 0.936 0.349

SQoL5 0.435 0.033 13.296 0.000

*HSCL-5*

STDYX Standardization

Two-Tailed

Estimate S.E. Est./S.E. P-Value

GEN BY

SQoL1 0.697 0.018 38.597 0.000

SQoL2 0.553 0.023 24.318 0.000

SQoL3 0.618 0.020 30.547 0.000

SQoL4 0.818 0.012 68.683 0.000

SQoL5 0.362 0.027 13.524 0.000

HSCL1 0.728 0.015 47.696 0.000

HSCL2 0.698 0.016 42.939 0.000

HSCL3 0.779 0.013 60.672 0.000

HSCL4 0.845 0.010 81.153 0.000

HSCL5 0.785 0.013 60.939 0.000

LIV BY

SQoL1 0.585 0.024 24.566 0.000

SQoL2 0.578 0.024 23.818 0.000

SQoL3 0.467 0.025 18.664 0.000

SQoL4 0.050 0.025 2.016 0.044

SQoL5 0.495 0.029 17.236 0.000

ANX BY

HSCL1 0.439 0.030 14.404 0.000

HSCL2 0.481 0.010 47.779 0.000

**Appendix B. (Bi)factor results based on a 4-factor model**

*Table B1. Estimates for 4-factor CFA model.*

RMSEA (Root Mean Square Error Of Approximation)

Estimate 0.083

90 Percent C.I. 0.076 0.089

Probability RMSEA <= .05 0.000

CFI/TLI

CFI 0.934

TLI 0.908

SRMR (Standardized Root Mean Square Residual)

Value 0.058

STDYX Standardization

Two-Tailed

Estimate S.E. Est./S.E. P-Value

F1 BY

SQoL1 0.948 0.010 95.202 0.000

SQoL2 0.774 0.017 46.760 0.000

F2 BY

SQoL3 0.898 0.012 72.987 0.000

SQoL8 0.608 0.022 27.581 0.000

SQol9 0.597 0.023 25.873 0.000

F3 BY

SQoL4 0.675 0.019 35.722 0.000

SQoL5 0.921 0.011 84.808 0.000

SQoL6 0.553 0.023 24.276 0.000

SQoL7 0.677 0.020 33.846 0.000

SQoL10 0.720 0.018 41.128 0.000

F4 BY

SQoL11 0.835 0.021 39.508 0.000

SQoL12 0.819 0.022 36.672 0.000

F2 WITH

F1 0.860 0.017 49.573 0.000

F3 WITH

F1 0.702 0.020 34.508 0.000

F2 0.643 0.026 24.615 0.000

F4 WITH

F1 0.629 0.025 24.956 0.000

F2 0.660 0.025 26.501 0.000

F3 0.418 0.031 13.434 0.000

SQoL4 WITH

SQoL10 0.567 0.025 23.032 0.000

Results for Bifactor (S-1) model with PHQ-2 defining the general factor and 4 additional specific factors (F1-F4): ECV_Gen_=.66, PUC=.84, ARPB=.12. Results for Bifactor (S-1) model with HSCL-5 defining the general factor and 4 additional specific factors (F1-F4): ECV_Gen_=.67, PUC=.89, ARPB=.14. Both models suggest essential unidimensionality.
